# Fungal Enzymes as Catalytic Tools for Polyethylene Terephthalate (PET) Degradation

**DOI:** 10.3390/jof7110931

**Published:** 2021-11-02

**Authors:** Seyedehazita Ahmaditabatabaei, Godfrey Kyazze, Hafiz M. N. Iqbal, Tajalli Keshavarz

**Affiliations:** 1 School of Life sciences, College of Liberal Arts and Sciences, University of Westminster, London W1W 6UW, UK; w1745896@my.westminster.ac.uk (S.A.); G.Kyazze@westminster.ac.uk (G.K.); 2 Tecnologico de Monterrey, School of Engineering and Sciences, Monterrey 64849, Mexico; hafiz.iqbal@tec.mx

**Keywords:** plastic, PET, PET-persistence, fungi, fungal enzyme, enzymatic degradation, by-products, enzyme engineering strategies

## Abstract

The ubiquitous persistence of plastic waste in diverse forms and different environmental matrices is one of the main challenges that modern societies are facing at present. The exponential utilization and recalcitrance of synthetic plastics, including polyethylene terephthalate (PET), results in their extensive accumulation, which is a significant threat to the ecosystem. The growing amount of plastic waste ending up in landfills and oceans is alarming due to its possible adverse effects on biota. Thus, there is an urgent need to mitigate plastic waste to tackle the environmental crisis of plastic pollution. With regards to PET, there is a plethora of literature on the transportation route, ingestion, environmental fate, amount, and the adverse ecological and human health effects. Several studies have described the deployment of various microbial enzymes with much focus on bacterial-enzyme mediated removal and remediation of PET. However, there is a lack of consolidated studies on the exploitation of fungal enzymes for PET degradation. Herein, an effort has been made to cover this literature gap by spotlighting the fungi and their unique enzymes, e.g., esterases, lipases, and cutinases. These fungal enzymes have emerged as candidates for the development of biocatalytic PET degradation processes. The first half of this review is focused on fungal biocatalysts involved in the degradation of PET. The latter half explains three main aspects: (1) catalytic mechanism of PET hydrolysis in the presence of cutinases as a model fungal enzyme, (2) limitations hindering enzymatic PET biodegradation, and (3) strategies for enhancement of enzymatic PET biodegradation.

## 1. Introduction

Plastics are synthetic materials of utmost importance in all modern societies. This is mainly because the robust attributes of plastic products evolved through time, including durability, weathering resistance, transparency, lightweight, low-price, high stability, and compact structural characteristics [1]. Undoubtedly, all these characteristics make plastics a vital entity for many domestic and industrial sectors [2]. Considering this high demand, over the past five decades, plastic-based products have become indispensable, increasingly replacing other products of domestic and industrial interests including products made partly or wholly from glass, metal, and wood. Over a longer time span, man-made synthetic plastic production has substantially increased up to three-fold in the last twenty-five years [3]. Regardless of their practical applied aspects, most of the used plastics have ended up as waste and accumulated in various environments [4]. As a result, plastic pollution is increasing at an alarming pace and is pervasive in different niches, including soil, sediments, agricultural land, marine, surface waters, water streams, and sludges [5,6]. Thus, plastic pollution has become a global and ubiquitous problem; urgent, holistic actions are essential to control and overcome serious damage to the environment and biological systems [5,6,7].

**Figure 1:**
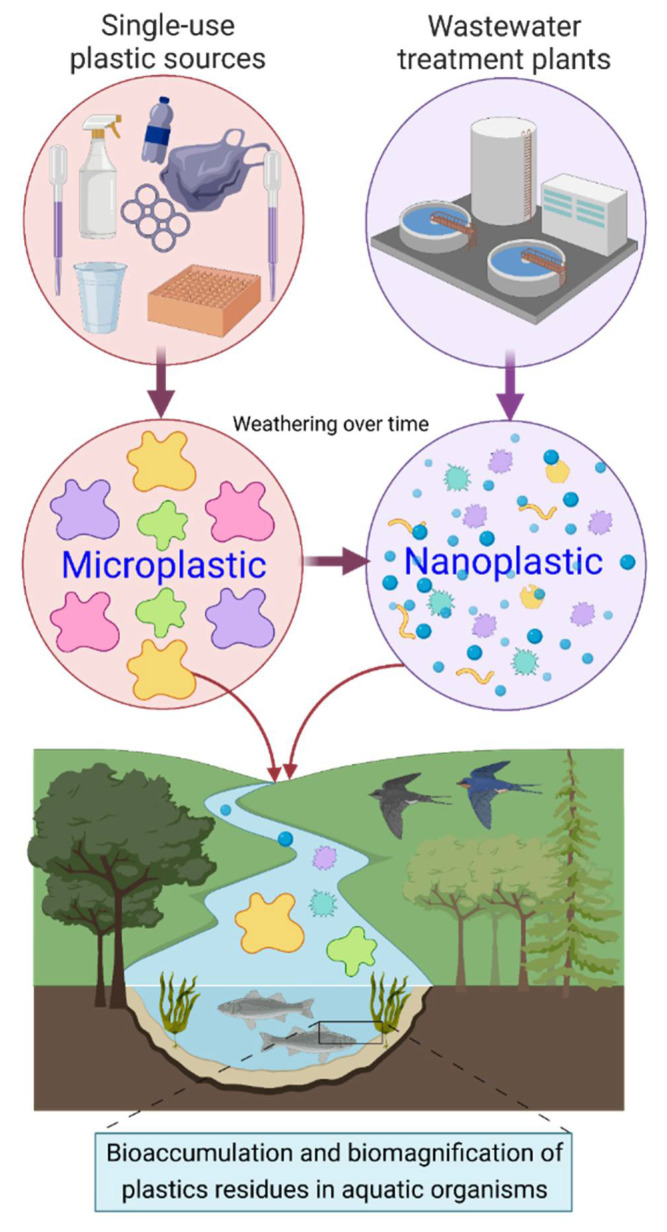
A schematic representation of various plastic sources, their ultimate weathering over time into micro-plastic and nano-plastic, and transportation into the aquatic environment. Created with BioRender.com and extracted under premium membership.

Accumulation of synthetic plastic debris at landfill sites and aqueous environments poses numerous detrimental effects on the entire ecosystem and its living beings [8,9,10]. Considering the plastic disposal in the aquatic environment alone, more than 9 million tons of plastic is dumped in oceans, which is expected to increase to double by 2025 [10,11]. Additionally, some chemicals that are added to plastics during their processing to improve their characteristics are toxic and hazardous to mammalian and marine life and affect chemical communication in aquatic ecosystems [12]. Furthermore, while present in the aquatic environment, plastics can attach to adjacent toxic contaminants such as heavy metals and organic pollutants creating hazardous entities. These entities, following several transformation processes, can enter various terrestrial or aquatic food chains and cause severe damage to the biota [13,14]. Eriksen et al. [15] estimated that there were approximately 269,000 tons of plastics submerged in surface waters globally. The presence of smaller plastic pieces in surface waters tends to result from the low degradability of larger pieces (macro-plastics) into smaller fragments considered as micro-plastics and/or nano-plastics. Figure 1 illustrates a schematic representation of various plastic sources, their ultimate weathering over time into micro-plastic and nano-plastic, and released into rivers, seas, and oceans. Considering the persistence/occurrence of plastics in the environment, plastics are divided into the following categories: (1) macro-plastics with particle size ranges >2.5 cm, (2) meso-plastics with particle size ranges from 2.5 cm to 5 mm, (3) micro-plastics with particle size ranges between 1 micron to 5 mm, and (4) nano-plastics with particle size ranges between 1 to 100 nm [16,17,18]. The reported effects of micro-plastic-based environmental pollutants on aquatic life include nearly 700 species, from tiny zooplankton to the largest whales. Out of the hundreds of aquatic species that are impacted adversely by micro-plastic pollution, 17% are IUCN (International Union for Conservation of Nature) red-listed species, and at least 10% have ingested plastics [19].

Most plastic wastes are due to the unnecessary or extensive consumption and discharge of plastics or plastic-based contaminating agents. Thus, it is vital to address global plastic pollution and minimize the adverse effects by taking holistic measures and strategies that encompass the entire plastics-based value chain. For instance, the key stakeholders and social actors, such as industrial sectors, governmental authorities, civil society members, academics, and basically, the whole population should step forward to take effective participation to address this issue [20,21].

## 2. Synthetic Plastics—Categories and PET

**Figure 2:**
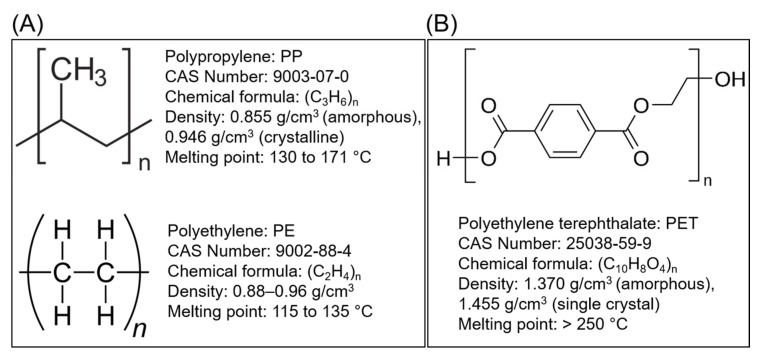
Structural and physicochemical characteristics; (**A**) category of non-hydrolysable plastics examples with a C–C backbone, and (**B**) category of hydrolysable plastics examples with a C–O backbone.

Considering the structural backbone, synthetic plastics have been broadly categorized into two groups, i.e., (1) plastics with a C–C backbone and (2) plastics with a C–O backbone (Figure 2). The first category of plastics is non-hydrolysable, and examples include polypropylene (PP) and polyethylene (PE), among others. These plastics contribute to 77% of the global market share. Furthermore, the minimally reactive C–C bonds in the backbone of polyesters are considered a significant obstacle to the biodegradation process [22]. The plastic materials in the second category with a C–O backbone are hydrolysable, and examples include polyethylene terephthalate (PET) and polyurethane (PU) among others and hold around 18% of the global market share [1,23,24]. Collectively, the global plastic market was valued at around $568.9 billion in 2019, which increased to $579.7 billion in 2020, and is expected to grow at a compound annual growth rate (CAGR) of 3.4% from 2021 to 2028 [25]. According to one estimate, until 2020, about 300 million tons (Mt) of plastic wastes was being produced annually, which has now escalated to 400 Mt annually. Further to this, the annual production of plastics is expected to double by 2035 (approx. 800 Mt) and reach 1600 Mt by 2050 [26,27]. Unfortunately, around 76% of the overall plastic production is handled as waste. Of this, 9% is recycled, 12% is incinerated, and 79% is landfilled or released to the environment [1,27].

PET is the most common single-use plastic among various synthetic plastics and is considered a thermoplastic polymer resin of the polyester family. PET is a clear, strong, and lightweight plastic that is widely used for packaging (Table 1) [28,29,30,31,32]. According to the British Plastic Federation (BPF), over 70% of the soft drinks in the global market are being packaged in PET bottles [33].

Antimony (Sb), a metalloid element, is used as a catalyst in the form of antimony trioxide (Sb_2_O_3_) or antimony triacetate in PET production. The WHO published a risk assessment for antimony in drinking water [34]. PET toxicity is typically associated with the leaching of Sb upon exposure to heat [35], thus it deserves careful consideration. Exposing PET to a thermal environment causes the leaching of antimony significantly, for example, into bottled water [36], possibly above US EPA maximum contamination levels [37]. As the presence of leached antimony in bottled water is a serious public health and safety concern, a detailed analysis of the published data on the presence, concentration, and leaching of PET is essential [38]. The persistence and toxicity risk aspects of PET additives are summarized in Table 2 [39,40,41,42,43,44,45,46,47,48,49,50].

Zheng et al. [51] observed that plastic polymers with pure carbon backbones are particularly resistant to most degradation methods. While this is often true, it is aromatic polymers that tend to be resistant to degradation, despite the presence of bonds that are typically readily hydrolyzed [52]. PET is a classic example of such a polymer, i.e., although the ester bond that is part of PET can be easily broken, PET is resistant to degradation due to the presence of a high ratio of aromatic terephthalate units [53]. This necessitates their removal from the environment. For this purpose, numerous methods are used, such as photo-oxidation, thermal degradation, chemical degradation, and biodegradation of PET [2,54,55,56]. However, each of these methods has its own merits and limitations. PET, as a polyester, is more resistant to biodegradation due to its ester bond group compared to other polymers. Several new studies on PET biodegradation by microbes, i.e., bacteria and their enzyme systems have been reported [2,56,57,58]. A plethora of literature is available on the bacterial enzyme-assisted degradation of PET [56,57,58]. However, little is published about fungal enzyme-mediated PET degradation. So far, there is a lack of robust fungal enzyme-mediated processes capable of efficiently mitigating the PET plastic-based contamination effectively and efficiently from ecosystems. For this, there is an urgent need for the development of sensitive and reliable detection systems that can be applied to the land- and -water-based plastic contaminants. This will enable the robust identification of plastic value chain hot spots that pose the most significant environmental problems. Thus, herein, an effort has been made to cover this literature gap by spotlighting the fungal strains and their potential enzyme systems as potential robust catalytic tools to degrade PET.

## 3. Fungal Enzyme-Mediated PET Degradation

Several microorganisms, including fungi and their unique enzyme systems, are capable of degrading PET (Table 3) [59,60,61,62,63,64,65,66,67,68,69,70,71,72,73,74,75,76]. Some fungal strains facilitate PET biodegradation into low molecular weight oligomers or monomers such as bis(2-hydroxyethyl)terephthalate (BHET) and mono(2-hydroxyethyl)terephthalate (MHET) [77]. The monomeric structural units of PET are linked by ester bonds, which many fungal hydrolytic enzymes can hydrolyze, e.g., esterases, lipases, and cutinases [59,60,61,73]. Esterases cleave the ester bonds (short-chain acyl ester) found in PET monomers and also facilitate the surface modification of the target PET [73]. Lipases are widely known for their catalytic hydrolysis of PET fabrics to some extent through enhancing their wettability, and the interfacial activation phenomenon characterizes them. Cutinases are lipolytic esterolytic enzymes with assertive catalytic behavior toward PET degradation [69]. Several fungal strains are significant sources of cutinases. For instance, cutinases from *Aspergillus oryzae*, *Aspergillus nidulans* [78], *Penicillium citrinum* [73], *Humicola insolens* [70], *Fusarium solani* [61,75], *Fusarium solani* pisi [76], and *Fusarium oxysporum* [64,65] have shown hydrolyzing activity toward low-crystallinity (*Ic*) PET.

Although the ester bond that is part of PET can be easily broken, PET is resistant to degradation due to the presence of a high ratio of aromatic terephthalate units [53]. The key factors that contribute to PET resistance to degradation include crystallinity, PET molecular weight, polymeric chain flexibility, surface hydrophobicity, and hydrolysis reaction temperature [79,80]. The stiffness of PET, due to the aromatic terephthalate building blocks, is a primary reason for PET’s low biodegradability. Moreover, a high ratio of aromatic terephthalate units in PET structure reduces chain mobility/flexibility and limits enzymatic degradation. However, a variety of fungi possess the potential to make PET more amenable to degradation through their enzyme system. So far, an array of fungal hydrolytic enzymes has been identified and deployed for various purposes [59,62,64,81]. However, limited studies have been performed on the PET degrading capacity of fungal hydrolytic enzymes and further use of PET monomers as a carbon source for enzyme secreting fungi. Thus, a thorough screening of fungal strains is crucial to identify the key enzymes, with high specific activity and efficiency, involved in plastic degradation at large and PET degradation in particular. Herein, we have reviewed comprehensively fungal enzymes capable of degrading PET obtained from different sources, including PET waste plastic bottles, PET woven fabric, PET films, PET powder, and flakes [59,62,64,81].

The effect of different crystallinity on the enzymatic degradation could be explained by the changes in the macromolecular aggregate structures of the polymer. Polymer molecules generally pack together in a non-uniform manner with a mixture of ordered regions (crystalline-like) and disordered domains (amorphous-like). In the amorphous domains, polymer chains are less densely packed than those in the crystalline domains. The PET containing a high percentage of amorphous domains is more prone to enzymatic degradation. The enzymatic hydrolytic reactions of PET are supposed to take place under the temperatures close to the glass transition temperature (*T_g_*) of PET (65~80 °C). Under such reaction conditions, the polymer chains in the amorphous PET domains can gain enough mobility to access the active sites of PET hydrolases [70,82]. Hence, it is supposed that faster PET degradation rates could be achieved by increasing the temperatures of the enzymatic hydrolysis reaction (for heat-tolerant enzymes) up to the glass transition temperature of PET [83]. Nevertheless, the high-crystallinity PET (30~40%) represents the amplest types of post-consumer plastic, and methods for lowering the crystallinity of PET to enhance the enzymatic degradation are of high interest [84]. The enzymatic degradation of PET is a heterogeneous catalytic process. However, the end products of the PET hydrolysis differ due to the reaction type, e.g., a catalytic reaction with or without additional supplementation of natural biosurfactants (i.e., hydrophobins), or synthetic surfactants (i.e., sodium lauryl sulfate), enzyme source and concentration, incubation temperature, and reaction period [83,85]. At the same time, robust strategies that assist in producing or fabricating thermo-stable PET hydrolyzing enzymes are required.

Tournier et al. [61] engineered PET depolymerases, including fungal *Fusarium solani* pisi cutinase (FsC) to break down and recycle plastic bottles. This improved enzyme-catalyzed PET depolymerization to 90% conversion into monomers in 10 h, with productivity of 16.7 g of terephthalate/L/h (200 g/kilogram of PET suspension, with an enzyme concentration of 3 milligrams/gram of PET). During enzymatic treatment, the surface pendant ester linkages on the PET can be easily hydrolyzed to polar hydroxyl and carboxylic groups, and further decompose to CO_2_ and water. The catalytic activities of cutinases from *Humilica insolens* (HiC) and *Fusarium solani* (FsC) using low-crystallinity (*lc*) and biaxially oriented (*bo*) PET films, as model substrates, were reported by Ronkvist et al. [70]. During 96 h degradation of *lc*PET films, FsC resulted in 5% film weight loss at 40 °C. Compared to FsC, HiC-catalyzed *lc*PET film hydrolysis at 70 °C resulted in 97 ± 3% weight loss in 96 h, corresponding to a loss in film thickness of 30 μm/day. As degradation of *lc*PET progressed, the crystallinity of the remaining film increased to 27% due to the preferential degradation of amorphous regions. The cutinases had about a 10-fold higher activity for the *lc*PET (7% crystallinity) than for the *bo*PET (35% crystallinity). Furthermore, for tested cutinases, analysis of aqueous soluble degradation products showed that they consist exclusively of TA and EG [70]. Polyesterase from *Penicillium citrinum* hydrolyzes both PET. From both plastic materials, bis-(2-hydroxyethyl)terephthalate and mono-(2-hydroxyethyl)terephthalate were released, while only low amounts of TA were liberated [73].

## 4. Catalytic Mechanism of Cutinases for PET Hydrolysis

**Figure 3:**
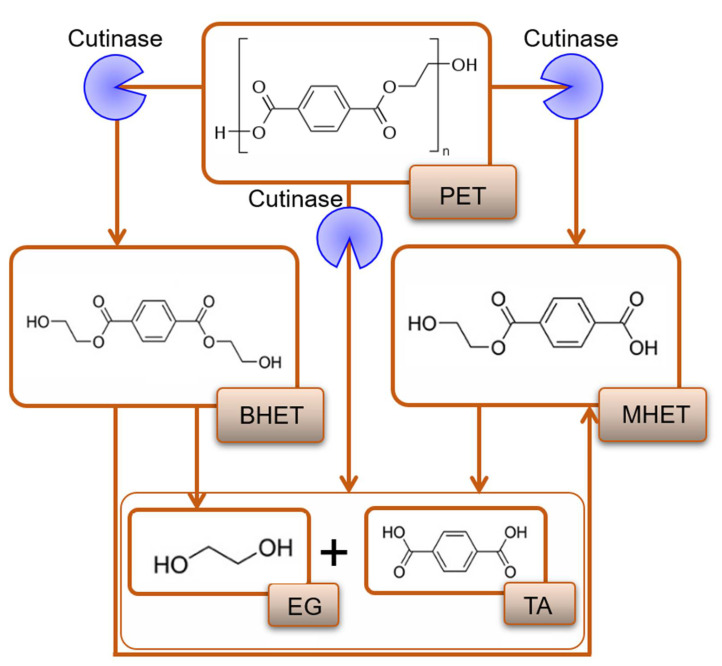
Cutinases mediated catalytic breakdown of PET into its subunits.

Cutinases mediated hydrolytic breakdown of PET into its subunits, e.g., BHET, MHET, TA, EG, have been identified as the water-soluble products of PET films and fibres [69]. In the presence of cutinase, PET hydrolysis is catalyzed by endo-type scission that cleaves internal ester bonds into end-products TA and EG (Figure 3) [86]. The active catalytic site of fungal cutinase from *Fusarium solani* includes Ser120, Asp175, and His188. During the initial reaction, the electrons from the oxygen of Ser120 react with the carbonyl group of PET, which leads to the formation of serine-terephthalate complex and ether compound. The fungal cutinase-assisted catalytic reaction causes the breakdown of PET into BHET, MHET, and TA [87]. The oxygen of the ether compound forms a covalent bond with the hydrogen of His188 residue of cutinase and forms EG. The oxygen from the Ser120 binds the hydrogen from the His188, and two molecules of cutinase and TA are released. The liberated cutinase molecules begin a new catalytic cycle [87].

## 5. Limitations Hindering Enzymatic PET Biodegradation

As discussed with the above examples, several fungal enzyme classes, i.e., esterases, lipases, and cutinases, have been identified with significant PET biodegradation potential in various routes, including direct or indirect catalytic breakdown (Figure 3). This led to the formation of PET oligomeric and monomeric units, i.e., BHET, MHET, TA, and EG [69,86,87]. However, some limitations lower or hinder the efficacy of the PET biodegradation process. For instance, high-crystalline PET has low catalytic turnover due to the limited approachability of the active sites. Furthermore, the high-crystalline PET has a higher *T*_g_ that causes kinetic uncertainty (enzyme saturation and unexpected alteration in its activity and stability) and loss of enzyme activity at the temperature above PETs *T*_g_ [88]. Thus, there is a need for high-temperature tolerant enzymes for efficient hydrolysis of high-crystalline PET. Inhibition by MHET or intermediate metabolites of the catalytic reaction process is another limitation in the enzymatic PET biodegradation [87,88,89]. Moreover, the formation of by-products during the biodegradation or hydrolysis process increases the acidity of the reaction solution [89], hence slowing down the reaction rate by inactivating the wild-type enzyme. Owing to these limitations, native wild-type enzymes do not function adequately. Improved catalytic performance can be accomplished through adopting various strategies, such as screening for high-temperature enzymes from hyperthermophilic strains, enzyme tailoring, genetic modification of the enzyme-producing strains, and/or deploying surfactants and additives. Each of these strategies that can assist in enhancing PET biodegradation is discussed in the following section with relevant examples.

## 6. Strategies to Enhance Enzyme-Based PET Biodegradation

### 6.1. Thermostable Enzymes

Hyperthermophile microbial strains with optimal activity and stability temperatures of >80 °C are important sources of high-temperature thermostable enzymes, so-called “thermo-zymes” (enzymes resistant to irreversible inactivation at high temperatures). Thermo-zymes are considered ideal candidates for catalytic processes that need to be operated at high temperatures. Several adaptive strategies can be followed to screen or synthesize enzymes giving them functionality in a high-temperature environment. Engineering high-temperature enzymes for robust catalytic transformation reactions are well covered in the literature [90,91,92,93], thus it is not the focus of this review. Screening thermophiles and engineered high-temperature enzymes, several other methods, such as the exploitation of ionic liquids, or deployment of suitable modifiers such as Ca^2+^, and various immobilization methods using robust support matrices have been adopted to increase the thermostability of PET hydrolases [94,95,96]. Thus, these thermophilic PET hydrolases could efficiently be used for PET biodegradation purposes. For example, the thermo-stability and catalytic activity of PET-degrading cutinase-like enzyme, Cut190, was boosted by high concentrations of Ca^2+^, which is essential for efficient enzymatic hydrolysis of amorphous PET [96]. The Cut190, a member of the lipase family, encompasses an α/β hydrolase fold and a Ser-His-Asp catalytic triad, thus hydrolyzing the inner block of PET [96].

### 6.2. Use of Surfactants and Additives

The catalytic turnover of enzyme-based reactions can be facilitated/boosted by using various surfactant molecules or surface-active additives in the enzymatic hydrolysis. Surfactants stabilize the enzymes, thereby effectively preventing enzyme denaturation during hydrolysis, which is a significant limitation of enzymatic PET biodegradation. The supplemented surfactant molecules tend to bind with the enzymes and alter the secondary and tertiary structures or flexibility of the enzyme, thereby shielding the enzyme kinetic properties [97]. Furthermore, the integration of surfactant molecules in the reaction medium can additionally improve the dispersibility of PET particles and thus may increase the accessibility of the substrate to enzymes. As mentioned earlier, the limited accessibility to substrate-binding active sites of the enzymes causes low activity for PET hydrolysis. This phenomenon may be ascribed to the hydrophobic force that prevents the enzyme from directly accessing the substrate [69,98]. The accessibility of the substrate to enzymes is very important as the presence of hydrophobic forces between the PET surface and reaction substrate is one of the significant limitations of the entire PET biodegradation process [99].

One considerable way to tackle this issue of surface hydrophobic/hydrophilic balance and substrate accessibility is the interfacial activation employing surfactant [69]. Hence, increasing the surface hydrophilization of PET near the substrate-binding region should promote cutinase-PET interactions, in the presence of surfactants, which is essential for its enzyme-assisted biodegradation. The ends of polymer chains on the PET surface are expected to protrude or form a loop [83]. Surface hydrophilicity could be increased through the hydrolysis of these loops to carboxylic acid and hydroxyl residues. The overall PET degradation can be further escalated by PET surface modification that is performed by the available microbial culture or its PET hydrolytic enzymes. PET surface properties can be improved by introducing surface-active additives to the PET surface to increase its hydrophilicity. In this context, the PET biodegradation potential of fungal cutinase from *Fusarium solani* pisi was induced by using various surfactants, including sodium dodecyl sulfate or sodium lauryl sulfate (SDS), Triton X-100, Tween 20, and sodium taurodeoxycholate (TDOC) at different concentrations in the presence of 20 mM Tris–HCl buffer of pH 8 [100]. Furthermore, various substrates, i.e., p-nitrophenyl butyrate (pNPB), p-nitrophenyl palmitate (pNPP), tributyrin, and triolein were also used to initiate the reaction. The results showed 73.65% PET biodegradation by *Fusarium solani* pisi cutinase that released soluble hydrolysis products, i.e., BHET, MHET, TA, and 1,2-ethylene-mono-terephthalate-mono(2-hydroxyethyl terephthalate) (EMT). The released hydrolysis products were detected and confirmed by LC-MS analysis [100]. Likewise, the incorporation of additive molecules, such as hydrophobins which are cysteine-rich surface-active proteins produced by filamentous fungi, has also been used to increase enzymatic PET hydrolysis [101,102,103]. Espino-Rammer et al. [101] tested two hydrophobins (HFBs), HFB4 and HFB7 of *Trichoderma* spp., to enhance the rate of enzymatic hydrolysis of PET. Both HFB4 and HFB7 displayed a dosage-dependent stimulation effect on PET hydrolysis by cutinase from *Humicola insolens*. Moreover, the simultaneous addition of *Humicola insolens* cutinase (final concentration, 0.2 mg/mL) and HFB4 (concentrations from 0.05 to 50 mg/liter) to PET resulted in stimulation of the cutinase activity. This was observed by measuring the released soluble hydrolysis products, TA and MHET [101].

### 6.3. Enzyme Tailoring and Genetic Modification

**Figure 4:**
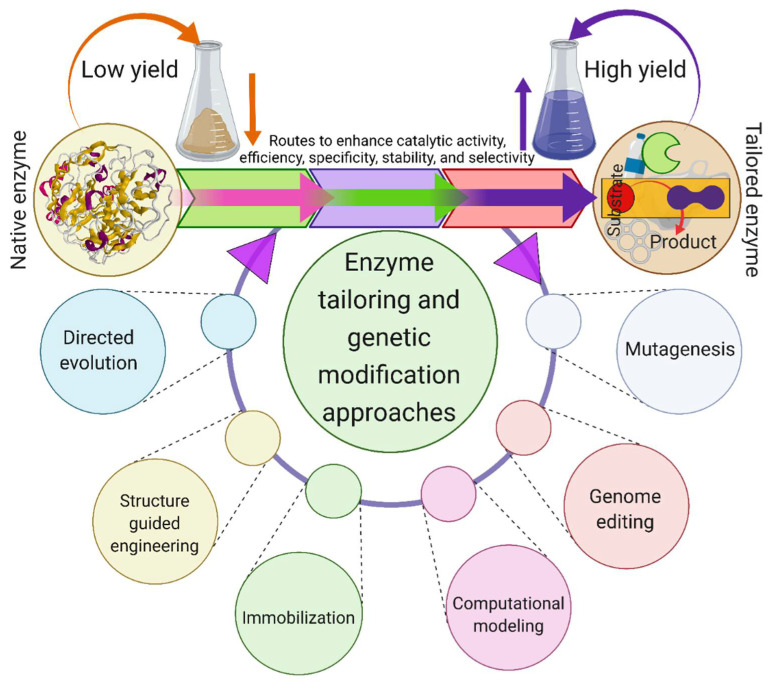
Protein engineering approaches to develop robust catalysts of interest with high catalytic activity, efficiency, specificity, stability, and selectivity, that could enhance PET biodegradation. Created with BioRender.com and extracted under premium membership.

The above-discussed shortcomings of enzymes can be overcome via enzyme tailoring and genetic modification practices. In addition, the tailored or genetically engineered enzyme-based catalysis offers multi-benefits, such as mild processing for complex and stable compounds, e.g., PET, and the capability to diminish reaction by-products or limit the generation of intermediate secondary products (that resist the enzymatic PET biodegradation) [22,91,93]. Moreover, the genomic modification settings/protocols that could enhance the PET biodegradation potential of enzymes, i.e., esterases, lipases, cutinases, and others need to be improved/modified. Several strategies, such as random mutagenesis and site-directed mutagenesis, genome editing, computational genomics and advanced computational modeling, structure-guided protein tailoring, and directed evolution are among the recent strategies that have been implemented to address the catalytic limitations of enzyme engineering (Figure 4) [22].

Several PET-degrading enzymes, including cutinases, have been immobilized using different support matrices. Nevertheless, these engineered enzyme-based catalytic systems have been used in other applications rather than in PET hydrolysis. Hence, there are limited reports on PET hydrolysis using immobilized fungal hydrolases/cutinases. For example, Nikolaivits et al. [104] engineered cross-linked enzyme aggregates (CLEAs) of cutinase from *Fusarium oxysporum*. As discussed above, cutinases have been reported for PET biodegradation, hence, this CLEAs-cutinase from *Fusarium oxysporum* could also be used for PET hydrolysis. In another study, Su et al. [105] used Lewatit VP OC 1600 (a macro-porous divinylbenzene-crosslinked methacrylate esters resin) as solid support to immobilize three cutinases, i.e., cutinase from *Aspergillus oryzae*, cutinase from *Humicola insolens* (a thermophilic fungus), and cutinase from *Thielavia terrestris*. Essentially, the solubility and rigidity of PET polymers increase and decrease, respectively, in organic solvents, thereby allowing easy access of the engineered enzymes to ester bonds of PET for efficient hydrolysis. Hence, this immobilized HiC could also be used for the hydrolysis of PET [89].

## 7. Conclusions and Future Considerations

The continuous and rapid development of the plastic industry has raised environmental issues globally, clearly evident from the massive PET waste bioaccumulation in the landfills, seas, and oceans. Traditional methods (incineration and landfilled) to recycle PET waste are still problematic because of the fatal consequence on aquatic animals and humans. Finding an effective and environment-friendly strategy for PET waste green recycling is in high demand. The discovery of new PET degrading microorganisms, mixed culture of fungal strains, and/or their genetically engineered robust enzyme systems, could be an effective strategy toward a green recycling scheme for PET waste. Moreover, studying their molecular mechanisms extensively via solving their crystal structure will widen this research area to move forward with industrial applications. The utilization of alternative and more dynamic chassis for enhancing PET biocatalysts production needs further investigations. The deployment of interdisciplinary and ground-breaking fungal strategies for PET biodegradation will reduce plastic waste pollution and help to clean the biosphere for a better tomorrow.

## Figures and Tables

**Table 1:** Polyethylene terephthalate (PET) packaging products based on end-user consumption. The global plastic consumption: 367 million tonnes, total PET packaging products consumption: 27 million tonnes in 2020 (7.4%). (Source: Data were extracted and calculated based on refs. [28,29,30,31,32]).

PET Packaging Products	Global Consumption in 2020 (Million Tonnes)
Water Bottles	7.02
Carbonated soft drink (CSD) bottles (e.g., Coca Cola, beers)	7.02
Other drinks (e.g., juices, milk)	4.86
Other bottles/containers in form of films and sheets	3.78
Food containers	2.43
Containers for non-food consumer products (e.g., cosmetics)	1.62

**Table 2:** Hazardous additives, present in PET products, and their effects.

Hazardous Additives	Chemical Formula	Chemical Structure	Toxic Effects	References
Bisphenol A (BPA)	$C_{15}H_{16}O_{2}$	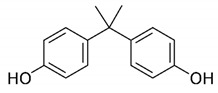	Female and male infertility Precocious puberty Breast cancer Prostate cancer Metabolic disorders including polycystic ovary syndrome (PCOS)	[39]
Bis (2-ethylhexyl) phthalate (DEHP)	$C_{24}H_{38}O_{4}$	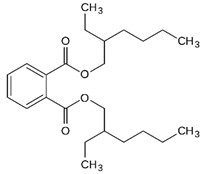	Cancer Reproductive system Stages of development Nerve system Immune system	[40]
Benzyl butyl phthalate (BBP)	$C_{19}H_{20}O_{4}$	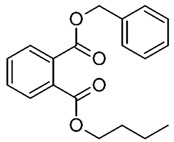	Decrease in thyroid hormone levels Endocrine system Stages of development Reproductive system	[41]
Lead chromate molybdate sulphate red	$\mathrm{Pb}\left(\mathrm{Cr},\mathrm{Mo},\;S\right)O_{4}$	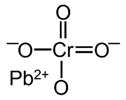	Cardiovascular system Respiratory system Gastrointestinal-liver Endocrine system Cancer Kidney damage Neurotoxic effects	[42]
Medium-chain chlorinated paraffins (MCCP)	$C_{14}H_{24}{\mathrm{Cl}}_{6}$ $C_{17}H_{29}{\mathrm{Cl}}_{7}$	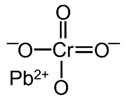	Skin dryness Adverse effects on aquatic life	[43]
Triclosan	$C_{12}H_{7}{\mathrm{Cl}}_{3}O_{2}$	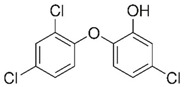	Thyroid hormones Reproductive system Breast cancer	[44]
Dibutyl phthalate (DBP)	$C_{6}H_{4}{\left({\mathrm{CO}}_{2}C_{4}H_{9}\right)}_{2}$	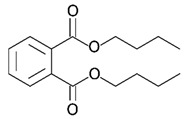	Effect on kidney Reproductive system Irritation of eyes, nose, throat, and skin	[42]
Diisobutyl phthalate (DiBP)	$C_{16}H_{22}O_{4}$	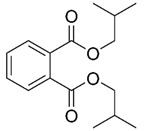	Reproductive system Developmental system Liver Kidney Possible triggering of cancer	[45]
Dicyclohexyl phthalate (DCHP)	$C_{20}H_{26}O_{4}$	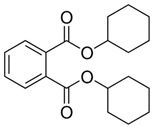	Reproductive system Cumulative anti-androgenic effect with other phthalates	[46]
Tris(2-chloroethyl)phosphate (TCEP)	$C_{6}H_{12}{\mathrm{Cl}}_{3}O_{4}P$	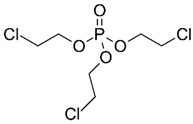	Possible impairment of fertility Adverse effects on aquatic organisms	[47]
1,3,5-Tris(oxiran-2-ylmethyl)-1,3,5-triazinane-2,4,6-trione (TGIC)	$C_{12}H_{15}N_{3}O_{6}$	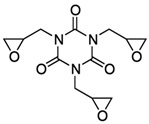	If swallowed If inhaled May cause genetic defects Serious eye damage	[42]
1,3,5-tris[(2S and 2R)-2,3-epoxypropyl]-1,3,5-triazine-2,4,6- (1H,3H,5H)-trione (β-TGIC)	$C_{24}H_{30}N_{6}O_{12}$	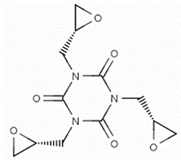	May cause genetic defects Harmful if swallowed Causes serious eye damage May cause damage to organs through prolonged or repeated exposure May cause an allergic skin reaction	[42]
Bisphenol S	$C_{12}H_{10}O_{4}S$	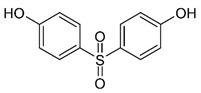	Obesity Metabolic disorders Possible triggering of cancer Reproductive defects Gestational diabetes Breast cancers	[48]
Benzophenone-3	$C_{14}H_{12}O_{3}$	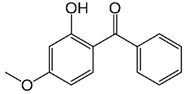	Allergic reactions Endocrine disruption Hirschsprung’s disease	[49]
Antimony trioxide	${\mathrm{Sb}}_{2}O_{3}$	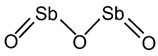	Possible triggering of lung cancer Reproductive system Kidney, liver, heart	[50]

**Table 3:** Fungal enzyme-mediated polyethylene terephthalate (PET) degradation.

Enzyme	Fungal Strain	PET Source	Percent PET-Degradation (Transformed Products)	Reference
Lipase and Cutinase	*Aspergillus tamarii* and *Penicillium crustosum*	PET films	TPA	[59]
Lipase	*Penicillium simplicissimum*	Post-consumer (PC)-PET	TPA, MHET and BHET	[60]
Cutinase	*Fusarium solani*	PET waste	90% conversion into monomers	[61]
NR	*Aspergillus sp.*	Waste Plastic bottles	22% weight loss after 6 weeks	[62]
Cutinase	*Fusarium solani*	Synthetic PET	EG	[63]
Cutinase	*Fusarium oxysporum*	PET woven fabric	TPA, MHET and BHET	[64]
Cutinase	*Fusarium oxysporum*	PET fabrics	NR	[65]
Cutinase	*Humicola insolens*	PET bottles	TPA, MHET and BHET	[66]
Lipase	*Candida antarctica*	PET bottles	TPA, MHET and BHET	[66]
Lipase	*Candida rugosa*	PET film	NR	[67]
Hydrolase	*Penicillium funiculosum*	PET film	0.21% weight loss	[68]
Lipase	*Thermomyces lanuginosus*	PET fabrics and films	TPA, BHET, MHET	[69]
Cutinase	*Fusarium solani*	PET fabrics and films	TPA, BHET, MHET,	[69]
Cutinase	*Humilica insolens*	NR	TPA, EG	[70]
Cutinase	*Fusarium solani*	NR	TPA, EG	[70]
Cutinase	*Fusarium solani*	PET fabrics	NR	[71]
Cutinase	*Fusarium solani*	PET fabrics	TPA	[72]
Polyesterase	*Penicillium citrinum*	PET pellets/fabrics	TPA, MHET, BHET and BA	[73]
Hydrolase	*Fusarium oxysporum* LCH I	PET fibers	TPA	[74]
Hydrolase	*Fusarium solani*	PET fibers	TPA	[74]
Hydrolase	*Fusarium solani*	Modified PET fabrics	NR	[75]
Cutinase	*Fusarium solani*	PET film	MHET	[76]

Abbreviations: PET—Polyethylene terephthalate; TPA—Terephthalic acid; MHET—Mono-(hydroxyethyl) terephthalate; BHET—Bis-(hydroxyethyl) terephthalate; EG—Ethylene glycol; NR—Not reported.

## Data Availability

Not applicable.
